# Analysis of a test system for the detection of SARS-CoV-2 in children attending school

**DOI:** 10.1093/eurpub/ckac131.052

**Published:** 2022-10-25

**Authors:** E Boietti, F Bert, G Lo Moro, S Barbaro, S Barbero, E Minutiello, T Sinigaglia, F Fagioli, R Siliquini

**Affiliations:** 1 Department of Public Health Sciences, University of Turin, Turin, Italy; 2 Regina Margherita Children’s Hospital, University of Turin, Turin, Italy

## Abstract

**Background:**

During the COVID-19 pandemic, in order to keep schools open and reduce SARS-CoV spreading, SARS-CoV-2 positive paediatric patients (PP) need to be isolated early. The aim of this study was to describe the appropriateness of school hot spot (HS) for SARS-CoV 2 testing based on open access of PP in a paediatric hospital in Turin, Italy.

**Methods:**

A cross-sectional study was performed between September 2020 and March 2021. The data collected included: date of swab execution, type of swab, execution setting of the swab, result of the swab, sex, age of PP and the mean value of the Rt (reproductive number) of pandemic in the Piedmont region. We collected data about PP from four different hospital settings (HS, Emergency department, day hospital and hospital wards) of Regina Margherita Children’s Hospital (Turin, Italy). We analyzed a sample of 13,283 PP (aged 0-19 years) testing for SARS-CoV-2. The main outcome was the likelihood of testing positive in different settings and in different age groups.

**Results:**

In Our sample, females were 45.8%. The median age was 6.8 years (IQR 3.0-11.2). The swabs executed in all the hospital settings had a lower likelihood of resulting positive compared with the school HS setting. Newborns below 3 months (adj OR 1.85, 95%CI 1.14 - 3) and patients aged between 11 and 13 years old (adj OR 1.32, 95%CI 1.07 - 1.63) reported a higher probability of a swab tested positive compared to adolescents. Instead, children aged between 3 months and 2 years (adj OR 0.77, 95%CI 0.61 - 0.96) and aged between 3 years and 5 years (adj OR 0.66, 95%CI 0.53 - 0.83) were less likely to result positive.

**Conclusions:**

We found a high prevalence of PP positive to the test for the detection of SARS-CoV-2 at the school hot spot compared with other settings. The open access modality to the nasopharyngeal swab was effective in identifying PP with COVID-19. Public health authorities should implement this testing modality in order to reduce SARS-CoV-2 infections in PP.

**Key messages:**

• Open access testing system to detect SARS-CoV-2 is important to do as many tests as possible to identify COVID-19 patients and isolate them in the pediatric population.

• The open access testing modality to detect COVID-19 patients saves time for doctors who, instead of carrying out the patient history, can devote themselves to other clinical activities.

